# Replacing Dairy Fat With Polyunsaturated and Monounsaturated Fatty Acids: A Food-Level Modeling Study of Dietary Nutrient Density and Diet Quality Using the 2013–16 National Health and Nutrition Examination Survey

**DOI:** 10.3389/fnut.2019.00113

**Published:** 2019-08-06

**Authors:** Colin D. Rehm, Adam Drewnowski

**Affiliations:** ^1^Department of Epidemiology & Population Health, Albert Einstein College of Medicine, Bronx, NY, United States; ^2^Center for Public Health Nutrition, University of Washington, Seattle, WA, United States

**Keywords:** food based dietary guidelines, dietary nutrient density, substitution modeling, healthy diet scores, NRF 9.3, HEI-2015, dairy, dietary fat

## Abstract

Recent dietary guidelines have become more food-based, as opposed to purely nutrient-based. By contrast, assessing the impact of dietary changes on chronic disease risk continues to rely on single-nutrient substitutions. To assess the real-world implications of a nutrient-for-nutrient swap, this study examined dietary nutrient density and healthy diet scores following removal of food sources of dairy fat from diets of 15,260 individuals age ≥4 y in the National Health and Nutrition Examination Survey (NHANES 2013–2016). Those foods were then replaced with foods containing a comparable amount of non-dairy polyunsaturated fatty acids (PUFA) and monounsaturated fatty acids (MUFA). The present food-level substitution model was based on 576 diverse eating patterns of US population subgroups. Diet quality measures were the Nutrient Rich Food (NRF 9.3) Index and the 2015-Healthy Eating Index (HEI-2015). Removing 5% of dietary energy from dairy fat led to lower levels of multiple micronutrients and to lower NRF 9.3 scores. These deficits were not remedied by the modeled replacements. Although swapping dairy fat for foods containing non-dairy MUFA/PUFA did alter the fatty acid ratios, the resulting food patterns were still significantly lower in some key micronutrients. Nutrient-based dietary guidance is prone to ignore the complexity of food patterns and the recommended dietary change may have unintended nutritional consequences.

## Introduction

Replacing 5% of energy intake from dairy fat with equivalent energy intake from polyunsaturated fatty acids (PUFA) has been linked to a 24% lower risk of cardiovascular disease (CVD) (1). This replacement was accomplished by exchanging different fat sources in regression analyses, substituting one regression coefficient for another (1). The results were taken as evidence in support of dietary guidelines to replace animal fats, including dairy fat, with PUFA for the prevention of CVD at the population level (1, 2).

The implementation of nutrient-based dietary guidelines has always been problematic (3). In the real world, people eat foods, not nutrients. Foods contain multiple nutrients that cannot be readily swapped for one another. For example, reducing dietary sodium while doubling the intakes of dietary potassium can be a challenge when many frequently eaten foods contain both (4–6).

In practice, a reduction of 5% of energy intake from dairy fat would mean a reduction in the consumption of the major food sources of dairy fat, that is to say milk and milk beverages, yogurt, and cheese, along with many mixed foods containing these ingredients (7–9). To complicate matters, habitual consumption patterns for milk, yogurt, butter, and cheese can vary widely across population subgroups (10, 11). Past studies on sociodemographic profiles of dairy consumers have pointed to sharp differences by gender, age group, income, education, and race/ethnicity (12–14). The type of dairy products and the amounts eaten can also depend on the food pattern chosen by the consumer. The recently issued United States Department of Agriculture (USDA) Healthy Food patterns recommended very different amounts for meat, poultry, fish, milk, and dairy depending on pattern type (15). All those factors are difficult to address in nutrient-based analyses, even after accounting for dietary intakes of fruit, vegetables, coffee, protein, and vegetable fat in multiple regression models (1).

The present aim was to assess the real-world implications of nutrient-driven dietary guidance. The primary outcome was diet quality following total or partial removal of foods containing dairy fat from the diet. The secondary outcome was diet quality following equal weight replacement of dairy fat with foods containing non-dairy PUFA and monounsaturated fat (MUFA). Selected micronutrients and composite measures of dietary nutrient density and a healthy diet score were the measures of diet quality. This food-level modeling was sensitive to the diversity of current dietary habits among multiple population subgroups in the United States.

## Methods

### Data Source and Population

Data for this project came from the two most recent cycles of the National Health and Nutrition Examination Survey (NHANES) corresponding to years 2013–2014 and 2015–2016. The NHANES is a nationally-representative diet and health survey of the US population and is the primary source of dietary surveillance data in the US. NHANES dietary intake data are used to inform the US dietary guidelines as well as other federal and state food and nutrition policies.

The present data analyses were based on the first 24 h recall, which is completed in-person by eligible participants. A single dietary recall is sufficient for measuring average intakes for populations, which was the primary goal of the present study (16). A second 24 h recall via telephone is collected as part of NHANES but was not used here. The NHANES dietary recall uses a multi-pass method and measures all foods consumed midnight-to-midnight in the day prior to data collection (17–19). Data were collected on mixed/composite foods such as stews, sandwiches and pasta dishes. The present analyses were based on 15,260 participants aged ≥4 y who completed a valid 24 h recall, as defined by National Center for Health Statistics (NCHS) staff.

### Identifying Dairy Fat

Foods containing dairy fats cannot be readily identified without combining a number of different data sources. This can be a challenge when identifying mixed foods containing dairy fat as one of many ingredients.

The Food and Nutrient Database for Dietary Studies (FNDDS) includes recipes for each individual food that can be linked to the USDA Standard Reference (SR) nutrient composition database (20) and the Food Patterns Equivalents database (21). This linkage allowed us to identify all dairy fat ingredients, including milk, cream, cheese, yogurt, ice cream, and butter, among many others. We queried the entire database for dairy-fat containing ingredients, allowing us to estimate the amount of dairy fat per 100 gram edible portion for each individual food.

The NHANES dietary data collection flags some consumed food items as combination foods (e.g., milk with cereal, coffee with milk, or a sandwich with cheese). This allowed us to identify any combination food containing dairy fat that was reported as consumed by NHANES participants. This information was then incorporated into the NHANES database that listed all foods consumed by individuals. Summing the amounts of dairy fat consumed from each food by each participant allowed us to estimate dairy fat consumption at the individual level. Summing the amounts of dairy fat over all foods consumed by all participants allowed us to estimate dairy fat consumption at the population level.

### Substitution Modeling Approach

All foods/beverages that were available for substitution modeling were identified in the nutrient composition database. Included were all foods consumed by NHANES participants, except those that contained dairy fat or those foods that had more saturated fat than unsaturated fat, in order to not replace food sources of dairy fat with other foods containing saturated fat.

In order to replace dairy fat with an approximately equivalent amount of non-dairy fat (PUFA and MUFA), we developed a multi-step substitution model. Briefly, foods containing dairy fat were replaced with foods containing PUFA and MUFA on a gram-per-gram basis. The replacement foods varied, depending on age, meal type, energy density, and percent energy from fat. We have previously used this approach in substitution modeling of 100% juices and whole fruit (22), ready to eat (RTE) cereals (23) and almonds and other tree nuts (24). Because energy density and percent energy from fat were included in the development of the replacement food, the ensuing substitution was approximately iso-caloric.

All foods were eligible to be included in the replacement model with a few notable exceptions. First, foods that contained dairy fat could not be included, nor could foods that contained more saturated fat than mono- and polyunsaturated fat. It was not possible to exclude foods that contained saturated fat altogether, as many foods that are high in MUFA/PUFA contain meaningful amounts of saturated fat. Conversely, many foods containing dairy fat also included MUFA/PUFA.

The frequency weighted nutrient profiles of the substitution foods were created based on age, meal type, dietary energy density, and percent of energy from fat. The four age groups were defined as 4–19 y, 20–39 y, 40–64 y, and 65+y. The four meal types were breakfast, snacks (including beverage only eating occasions), lunch/dinner, or dessert. Food energy density (in kcal/100 g) was classified into 6 categories, defined by weighted consumption at <10th percentile [<49.8 kcal/100 g], 10–24th percentile [49.8–70.6], 25–50th percentile [70.7–151.4], 50–74th percentile [151.5–235.5], 75–90th percentile [235.6–308.7] and 90th+ percentile [308.8–682]). Percent energy from fat was likewise classified into 6 categories, defined as: <10th percentile [0.7–16.3%E], 10–24th percentile [16.4–24.8%E], 25–50th percentile [24.9–38.3%], 50–74th percentile [38.4–48.2%], 75–90th percentile [48.3–63.4%], and 90th+ percentile [63.5–97.8%].

Food patterns of the NHANES population sample were thus split by age (4 categories), meal type (4 categories), dietary energy density (6 categories), and percent dietary fat (6 categories) for a total of 576 patterns. All foods consumed by NHANES participants within each of these 576 subgroups were then identified. Food based modeling needs to account for the fact that foods that contain PUFA/MUFA are not eaten in equal amounts by all population subgroups and their consumption can also vary, depending on age, meal type or dietary energy density. The substitution foods were category specific, and their nutrient profiles were weighted moreover by the consumption of each food within that subgroup. This means that the PUFA/MUFA containing foods that were frequently eaten by a given subgroup were weighted more heavily in the substitution model.

To illustrate this very detailed approach, the top-weighted foods in the lunch/dinner meal for the 40–64 y age group in the third sextile of energy density and second sextile of percent energy from fat were white rice made with oil, Spanish rice with added fat, brown rice made with oil, rice pilaf, Lo Mein noodles without meat, beef stew with potatoes and vegetables and gravy, and stewed chicken breast with the skin not eaten, among approximately 240 unique foods. The composite nutrient values per gram for the MUFA/PUFA foods for that group were then weighted based on survey weights and frequency of consumption.

Two substitution models were implemented. Model 1 removed all dairy fat (no limit) and replaced it with the weighted and subgroup-specific substitution foods. Model 2 removed up to 5% of energy from dairy fat for each person and replaced it with subgroup-specific substitution foods. For individuals consuming more than 5% of fat energy from dairy, all foods eaten on that day were randomized and the food contributing >5% of fat energy was replaced up to but not exceeding 5% for that individual. The randomization was necessary since otherwise some meals may systematically be more prone to being replaced (e.g., breakfast), which could bias the results.

### Dietary Nutrient Density

Because the models were only approximately iso-caloric, all dietary outcomes were energy-adjusted, with the exception of total energy, which is included in the tables for comparison purposes. The primary outcomes of interest were total fat, saturated fat, polyunsaturated fat, monounsaturated fat, carbohydrates, added sugars, and a number of micronutrients/minerals including calcium, vitamin D, fiber, potassium, vitamin A, and B-vitamins.

### Healthy Diet Scores

The Nutrient Rich Foods (NRF) index was the principal measure of diet quality (25, 26). Its development and validation with respect to other measures of diet quality and long term health outcomes have been described in the literature (27–29). The present NRF 9.3 variant applied to total diets was based on nine qualifying nutrients (NR) and three nutrients to limit (LIM), using a new approach to calculate the LIM components to be consistent with other measures of diet quality. Reference daily values (DVs) were based on standards issued by the US Food and Drug Administration (FDA) and the Institute of Medicine (26). The qualifying nutrients and standard reference amounts were as follows: protein (50 g), fiber (28 g), vitamin A (900 RAE), vitamin C (90 mg), vitamin D (20 mcg), calcium (1,300 mg), iron (18 mg), potassium (4,700 mg), and magnesium (420 mg). In the NR calculation, each daily nutrient intake was adjusted for 2,000 kcal and expressed in percentage of DV. Percent DVs for nutrients were truncated at 100, so that an excessively high intake of one nutrient could not compensate for the dietary inadequacy of another. The NR score is then calculated as the sum of the NR components, the minimum score is 0 and the maximum score is 900.

Unlike the NR component, the LIM component that includes added sugars, sodium and saturated fat, are calculated in a manner similar to the HEI-2015 (30). For each nutrient, values above the maximum threshold received a maximum number of limiting points (26%E for added sugars, 16%E for saturated fat and 2,000 mg for sodium); intakes below the minimum threshold received zero limiting points (6%E for added sugars, 8% for saturated fat, and 1,100 mg sodium). Intakes between the minimum and maximum thresholds were calculated on a proportional basis. For example, an individual consuming 12%E from saturated fat would earn 0.5 limiting points. The minimum LIM score is 0 (optimal) and the maximum is 300 (sub-optimal).

The NRF 9.3 was calculated as follows:

(1) $$\begin{array}{r} \text{NRF }9.3=\left(\text{NR}-\text{LIM}\right) \end{array}$$

The development and validation of the NRF family of nutrient density scores are all well-documented in the literature (28, 29). In recent iterations, vitamin D replaced vitamin E. Fiber, vitamin D, calcium, magnesium, and potassium were all identified in the 2010 Dietary Guidelines for Americans as nutrients of concern (31). The NRF score was adjusted for energy intake, similar to other diet quality scores, including the Healthy Eating Index-2015 (30).

The 2015-Healthy Eating Index (HEI-2015) was used as an additional summary measure of diet quality and specifically measures adherence to the 2015–2020 Dietary Guidelines for Americans. The HEI-2015 is an energy adjusted summary measure of diet quality based on the intake of nine food groups/nutrients to encourage including total fruits, whole fruits, total vegetables, greens and beans, whole grains, dairy, total protein foods, seafood and plant protein, and fatty acids ratio (ratio of unsaturated fat to saturated fat), and four food groups/nutrients to discourage, including refined grains, sodium, added sugars and saturated (30). The USDA Food Patterns Equivalents Database (FPED) was used to estimate intakes of each food group component (21). The HEI-2015 is scaled 0 to 100, with higher scores indicating increased adherence to dietary guidelines and vice versa (30, 32).

### Analytical Approach

For each dietary outcome of interest, the population mean and corresponding 95% confidence interval (CI) of energy-adjusted intake was calculated. Given the large sample size and statistical precision of the dietary outcomes, even small differences in observed and modeled intakes would be statistically significant. For these reasons, a 10% relative change comparing observed and modeled intakes was used as the threshold for statistical significance and all *p*-values used this threshold. Results are presented for the overall population and also limited to consumers of dairy fat. An alpha-level of 0.05 was used for all analyses. Model implementation and statistical analyses were performed using Stata 13.1 (College Station, TX).

The analysis of publicly available de-identified data is not considered human subjects research by the University of Washington or Albert Einstein College of Medicine.

## Results

Table 1 shows population characteristics and mean consumption of energy from dairy fat by sociodemographic variables. Data are for a representative sample of the US population (*n* = 15,260) from 2013 to 2016 NHANES cycles. Dairy fat accounted for 5.6% of daily dietary energy on the population-level. Dairy fat consumption did not vary by gender, and tended to decline with age. Dairy fat consumption was not associated with education or income. Non-Hispanic whites consumed most dairy fat (6.1% energy); non-Hispanic Asians consumed the least (3.8%).

**Table 1:** Sample description and mean consumption of energy from dairy fat overall and by socio-demographic group, United States 2013–2016.

	***N***	**Weighted percent**	**Mean dairy fat, %**
Total	15,260	–	5.6
**Age**			
4–8 y	1,685	6.7	6.8
9–13 y-	1,658	6.7	6.4
14–19 y	1,853	8.7	5.8
20–39 y	3,433	28.2	5.3
40–59 y	3,380	28.4	5.2
≥60 y	3,251	21.4	5.6
**Gender**			
Male	7,455	49.1	5.5
Female	7,805	50.9	5.6
**Race/ethnicity**			
Non-Hispanic white	5,355	62.1	6.1
Hispanic	4,432	16.9	5.4
Non-Hispanic black	3,307	11.9	4.1
Non-Hispanic Asian	1,495	5.4	3.8
Other/mixed race	671	3.7	5.0
**Family income to poverty ratio**			
<1.3 (lower income)	5,155	25.5	5.5
1.3–1.84	1,842	11.0	5.5
1.85–2.99	2,471	18.2	5.8
≥3.00 (higher income)	4,574	45.4	5.5
Missing	1,218	–	5.4
**Education (age** **≥** **25 y)**			
<High school	2,039	14.3	5.5
High school/equivalent	2,018	21.0	5.5
Some college	2,699	31.6	5.8
≥College	2,458	33.1	5.5

### Substitution Effects on Nutrient Intakes

Since dairy fat accounted for about 5% of daily energy at the population level, removing 5% of energy from dairy fat effectively meant removing 65% of dairy products from the diet. Table 2 shows the observed and modeled dietary intakes for the NHANES sample, including dairy consumers and non-consumers. Model 1 removed all dairy fat; Model 2 removed up to 5% of energy from dairy fat for each individual. In Model 1, dairy fat was reduced to 0% of energy, in Model 2 it was reduced to 1.7%. Saturated fat was significantly reduced in both models.

**Table 2:** Observed and modeled dietary intakes for the total sample following removal of dairy fat, NHANES, 2013–2016.

	**Mean (95% Confidence Intervals [CI])**		
	**Observed**	**Model 1**^a^** (remove)**	**Model 2**^b^** (remove)**
**Total population (*****n*** **=** **15,260)**			
**Macronutrients**			
Energy, kcal	2,080 (2,055, 2,105)	1,454 (1,435, 1,474)^***^	1,580 (1,558, 1,601)^***^
Protein, %E	15.8 (15.5, 16)	14.2 (13.9, 14.5)	14.7 (14.4, 15)
Total fat, %E	34.7 (34.4, 35)	31.5 (31.2, 31.8)	32.5 (32.1, 32.8)
Saturated fat, %E	11.4 (11.2, 11.5)	8.7 (8.6, 8.9)^***^	9.6 (9.4, 9.7)^***^
PUFA, %E	8.0 (7.9, 8.1)	8.3 (8.2, 8.5)	8.2 (8.1, 8.4)
MUFA, %E	12 (11.9, 12.1)	11.4 (11.2, 11.5)	11.6 (11.4, 11.7)
Dairy fat, %E	5.6 (5.4, 5.8)	0 (0, 0)^***^	1.7 (1.6, 1.9)^***^
Carbohydrate, %E	48.4 (48, 48.8)	52.3 (51.8, 52.8)	51.1 (50.6, 51.5)
Added sugar, %E	13.4 (13.1, 13.8)	16 (15.5, 16.4)^***^	15.2 (14.8, 15.6)^***^
**Micronutrients**			
Calcium, mg	965 (949, 981)	715 (700, 730)^***^	784 (772, 796)^***^
Vitamin D, mcg	4.8 (4.7, 5)	2.7 (2.5, 2.9)^***^	3.4 (3.2, 3.6)^***^
Fiber, g	16.6 (16.2, 16.9)	18 (17.6, 18.5)	17.5 (17.1, 17.9)
Potassium, mg	2,547 (2,510, 2,584)	2,618 (2,558, 2,679)	2,577 (2,535, 2,620)
Vitamin A, mcg	637 (618, 656)	490 (465, 516)^***^	531 (510, 553)^***^
Thiamin, mg	1.6 (1.6, 1.6)	1.5 (1.5, 1.5)	1.5 (1.5, 1.6)
Riboflavin, mg	2.1 (2.1, 2.1)	1.8 (1.7, 1.9)^**^	1.9 (1.8, 1.9)^*^
Niacin, mg	25.1 (24.8, 25.4)	25.8 (25.2, 26.3)	25.6 (25.1, 26)
Vitamin B6, mg	2.1 (2, 2.1)	2.1 (2, 2.1)	2.1 (2, 2.1)
Vitamin B12, mcg	4.8 (4.7, 4.9)	3.6 (3.5, 3.7)^***^	4 (3.9, 4.1)^***^
Folic acid mcg	390 (383, 398)	369 (362, 377)	376 (368, 383)

a Model 1 removes all dairy fat. Model 2 removes up to 5% dairy fat for each individual.

b Asterisks indicate statistical significance comparing whether Modeled changes are a 10% change from observed diets;

*** p < 0.001;

** 0.001 <

p < 0.01;

* 0.01 < p < 0.05.

The removal of dairy fat led to dietary patterns that were significantly lower in energy but were significantly higher in added sugars. There were significant reductions in modeled daily intakes of calcium, vitamin D, vitamin A, riboflavin, and vitamin B12. The same reductions in energy and key nutrients were observed in Model 1 and 2.

Table 3 shows the observed and modeled dietary intakes for dairy fat consumers only (*n* = 12,831). following the removal of dairy fat from the diet. Removing dairy fat led to the expected reduction in saturated fat intakes. Calories were significantly lower, as were calcium, vitamin D, vitamin A, riboflavin, and vitamin B12. By contrast, added sugars were higher. The same reductions in energy and some key micronutrients were observed in Model 1 and 2.

**Table 3:** Observed and modeled dietary intakes for the dairy fat following the removal of dairy fat, NHANES 2013–2016.

	**Mean (95% Confidence Intervals [CI])**		
	**Observed**	**Model 1^a^ (remove)**	**Model 2^b^ (remove)**
**Consumers of dairy fat only (*****n*** **=** **12,831)**			
**Macronutrients**			
Energy, kcal	2,133 (2,108, 2,157)	1,397 (1,379, 1,416)^***^	1,545 (1,524, 1,565)^***^
Protein, %E	15.7 (15.5, 16)	13.9 (13.6, 14.2)^**^	14.5 (14.2, 14.8)
Total fat, %E	35.1 (34.7, 35.4)	31.3 (30.9, 31.7)^**^	32.5 (32.1, 32.8)
Saturated fat, %E	11.8 (11.6, 11.9)	8.7 (8.6, 8.8)^***^	9.7 (9.5, 9.8)^***^
PUFA, %E	7.9 (7.8, 8)	8.3 (8.2, 8.4)	8.2 (8, 8.3)
MUFA, %E	12.1 (11.9, 12.2)	11.3 (11.2, 11.5)	11.6 (11.4, 11.7)
Dairy fat, %E	6.5 (6.3, 6.7)	0 (0, 0)^***^	2 (1.9, 2.2)^***^
Carbohydrate, %E	48.1 (47.7, 48.5)	52.7 (52.2, 53.2)	51.2 (50.7, 51.7)
Added sugar, %E	13.2 (12.9, 13.5)	16.1 (15.7, 16.5)^***^	15.2 (14.8, 15.7)^***^
**Micronutrients**			
Calcium, mg	1,014 (998, 1,030)	720 (702, 737)^***^	801 (787, 814)^***^
Vitamin D, mcg	5.1 (5, 5.2)	2.6 (2.4, 2.8)^***^	3.4 (3.2, 3.6)^***^
Fiber, g	16.5 (16.2, 16.9)	18.2 (17.8, 18.7)	17.6 (17.2, 18)
Potassium, mg	2,553 (2,517, 2,590)	2,637 (2,568, 2,706)	2,589 (2,543, 2,634)
Vitamin A, mcg	657 (638, 675)	484 (457, 511)^***^	532 (510, 554)^***^
Thiamin, mg	1.6 (1.6, 1.6)	1.5 (1.5, 1.5)	1.5 (1.5, 1.6)
Riboflavin, mg	2.2 (2.1, 2.2)	1.8 (1.7, 1.9)^***^	1.9 (1.9, 1.9)^*^
Niacin, mg	24.7 (24.4, 25.1)	25.5 (24.9, 26.1)	25.3 (24.8, 25.8)
Vitamin B6, mg	2.1 (2, 2.1)	2.1 (2, 2.1)	2.1 (2, 2.1)
Vitamin B12, mcg	4.9 (4.8, 5)	3.5 (3.4, 3.6)^***^	4 (3.9, 4.1)^***^
Folic acid mcg	392 (385, 398)	367 (360, 374)	374 (367, 382)

a Model 1 removes all dairy fat. Model 2 removes up to 5% dairy fat for each individual.

b Asterisks indicate statistical significance comparing whether Modeled changes are a 10% change from observed diets;

*** p < 0.001;

** 0.001 < p < 0.01;

* *0.01 < p < 0.05*.

Table 4 shows observed and modeled dietary intakes following the swapping of dairy fat for non-dairy fat for the total NHANES sample. Energy intakes were the same, given that the fat swap was approximately iso-caloric. Dairy fat was eliminated; saturated fat declined from 11.4 to 9.2% of energy, and PUFA increased from 8.0 to 9.4% of energy. The swapping of dairy fat for PUFA/MUFA containing foods resulted in modeled food patterns that were significantly lower in calcium, vitamin D, vitamin A, riboflavin, niacin, and vitamin B12. Generally similar results were obtained with Model 1 and 2. While a single 24 h recall cannot be used to estimate the exact proportion of the population meeting or not meeting a specific dietary requirement, the proportion of the population that consumed 1,000 mg/d or more of calcium decreased from 40.6% in observed diets to only 13.8% in Model 1 and 25.1% in Model 2.

**Table 4:** Observed and modeled dietary intakes for the total sample following the replacement of dairy fat with PUFA/MUFA, NHANES, 2013–2016.

	**Mean (95% Confidence Intervals [CI])**		
	**Observed**	**Model 1^a^ (swap)**	**Model 2^b^ (swap)**
**Total population (*****n=*** **15,260)**			
**Macronutrients**			
Energy, kcal	2,080 (2,055, 2,105)	2,113 (2,087, 2,139)	2,099 (2,073, 2,124)
Protein, %E	15.8 (15.5, 16)	16.1 (15.9, 16.4)	16.0 (15.8, 16.2)
Total fat, %E	34.7 (34.4, 35)	34.7 (34.4, 35)	34.7 (34.4, 35)
Saturated fat, %E	11.4 (11.2, 11.5)	9.2 (9.1, 9.3)^***^	10.2 (10.1, 10.3)^*^
PUFA, %E	8.0 (7.9, 8.1)	9.4 (9.3, 9.6)^***^	8.8 (8.7, 8.9)^*^
MUFA, %E	12 (11.9, 12.1)	12.8 (12.7, 13)	12.5 (12.4, 12.6)
Dairy fat, %E	5.6 (5.4, 5.8)	0 (0, 0)^***^	2.6 (2.4, 2.7)^***^
Carbohydrate, %E	48.4 (48, 48.8)	48.2 (47.8, 48.6)	48.2 (47.9, 48.6)
Added sugar, %E	13.4 (13.1, 13.8)	12.7 (12.4, 13)	13.0 (12.6, 13.3)
**Micronutrients**			
Calcium, mg	965 (949, 981)	668 (659, 678)^***^	796 (785, 807)^***^
Vitamin D, mcg	4.8 (4.7, 5)	3.5 (3.4, 3.7)^***^	4 (3.9, 4.1)^***^
Fiber, g	16.6 (16.2, 16.9)	18.2 (17.8, 18.6)	17.6 (17.2, 18)
Potassium, mg	2,547 (2,510, 2,584)	2,599 (2,563, 2,634)	2,575 (2,541, 2,609)
Vitamin A, mcg	637 (618, 656)	564 (547, 580)^***^	590 (573, 608)^***^
Thiamin, mg	1.6 (1.6, 1.6)	1.6 (1.6, 1.6)	1.6 (1.6, 1.6)
Riboflavin, mg	2.1 (2.1, 2.1)	1.8 (1.8, 1.8)^***^	1.9 (1.9, 1.9)
Niacin, mg	25.1 (24.8, 25.4)	27.3 (27, 27.7)^**^	26.3 (26, 26.7)
Vitamin B6, mg	2.1 (2, 2.1)	2.2 (2.1, 2.2)	2.1 (2.1, 2.1)
Vitamin B12, mcg	4.8 (4.7, 4.9)	4.2 (4.2, 4.3)^**^	4.4 (4.4, 4.5)
Folic acid mcg	390 (383, 398)	388 (381, 394)	387 (380, 394)

a Model 1 replaces all dairy fat. Model 2 replaces up to 5% dairy fat for each individual.

b Asterisks indicate statistical significance comparing whether Modeled changes are a 10% change from observed diets;

*** p < 0.001;

** 0.001 < p < 0.01;

* 0.01 < p < 0.05.

Table 5 shows observed and modeled dietary intakes following swapping dairy fat for PUFA/MUFA containing foods for consumers of dairy fat (*n* = 12,831). Energy intakes were the same, as expected. Dairy fat was eliminated; saturated fat declined from 11.8 to 9.2% of energy, and PUFA increased from 7.9 to 9.6% of energy. The swapping of dairy fat for PUFA/MUFA containing foods resulted in food patterns that were significantly lower in calcium, vitamin D, vitamin A, riboflavin, and vitamin B12. Substantially similar results were obtained with Model 1 and 2. Among dairy fat consumers, the proportion of persons consuming >1,000 mg of calcium dropped from 44.7 to 13.0% in Model 1 and 26.6% in Model 2.

**Table 5:** Observed and modeled dietary intakes for dairy fat consumers following the replacement of dairy fat with PUFA/MUFA, NHANES, 2013–2016.

	**Mean (95% Confidence Intervals [CI])**		
	**Observed**	**Model 1^a^ (swap)**	**Model 2^b^ (swap)**
**Consumers of dairy fat only (*****n=*** **12,831)**			
**Macronutrients**			
Energy, kcal	2,133 (2,108, 2,157)	2,172 (2,147, 2,196)	2,155 (2,130, 2,179)
Protein, %E	15.7 (15.5, 16)	16.2 (15.9, 16.4)	16 (15.8, 16.2)
Total fat, %E	35.1 (34.7, 35.4)	35.1 (34.7, 35.4)	35.1 (34.7, 35.4)
Saturated fat, %E	11.8 (11.6, 11.9)	9.2 (9.1, 9.3)^***^	10.4 (10.3, 10.5)^***^
PUFA, %E	7.9 (7.8, 8)	9.6 (9.5, 9.7)^***^	8.8 (8.7, 8.9)^***^
MUFA, %E	12.1 (11.9, 12.2)	13.1 (12.9, 13.2)	12.6 (12.5, 12.8)
Dairy fat, %E	6.5 (6.3, 6.7)	0 (0, 0)^***^	3.0 (2.9, 3.2)^***^
Carbohydrate, %E	48.1 (47.7, 48.5)	47.9 (47.4, 48.3)	47.9 (47.5, 48.3)
Added sugar, %E	13.2 (12.9, 13.5)	12.3 (12, 12.6)	12.6 (12.3, 12.9)
**Micronutrients**			
Calcium, mg	1,014 (998, 1,030)	666 (656, 676)^***^	815 (804, 827)^***^
Vitamin D, mcg	5.1 (5, 5.2)	3.6 (3.4, 3.7)^***^	4.1 (4, 4.3)^***^
Fiber, g	16.5 (16.2, 16.9)	18.4 (18.1, 18.8)	17.7 (17.4, 18.1)
Potassium, mg	2,553 (2,517, 2,590)	2,614 (2,580, 2,648)	2,586 (2,553, 2,620)
Vitamin A, mcg	657 (638, 675)	571 (555, 586)^***^	602 (585, 619)
Thiamin, mg	1.6 (1.6, 1.6)	1.6 (1.6, 1.6)	1.6 (1.6, 1.6)
Riboflavin, mg	2.2 (2.1, 2.2)	1.8 (1.8, 1.8)^***^	2 (1.9, 2)
Niacin, mg	24.7 (24.4, 25.1)	27.3 (27, 27.7)	26.2 (25.8, 26.5)
Vitamin B6, mg	2.1 (2, 2.1)	2.1 (2.1, 2.1)	2.2 (2.1, 2.2)
Vitamin B12, mcg	4.9 (4.8, 5)	4.2 (4.2, 4.3)^***^	4.5 (4.4, 4.5)
Folic acid mcg	392 (385, 398)	389 (383, 395)	388 (382, 394)

a Model 1 replaces all dairy fat. Model 2 replaces up to 5% dairy fat for each individual.

b Asterisks indicate statistical significance comparing whether Modeled changes are a 10% change from observed diets;

*** p < 0.001.

### Food-Level Substitution Effects on Healthy Diet Scores

The NRF 9.3 nutrient density score is nutrient based; it contains qualifying nutrients as well as nutrients to limit: saturated fat, added sugar and sodium. Table 6 shows observed and modeled healthy diet scores following the removal of dairy fat. Shown are mean NRF 9.3 and HEI-2015 overall and component scores and the data are for the total NHANES sample and for dairy fat consumers.

**Table 6:** Observed and modeled mean NRF 9.3 and HEI-2015 overall and component scores following the removal of dairy fat, NHANES 2013–2016.

	**Mean (95% Confidence Intervals [CI])**		
	**Observed**	**Model 1^**a**^ (remove)**	**Model 2^**b**^ (remove)**
**Total population (*****n=*** **15,260)**			
NRF 9.3	391 (385, 396)	314 (309, 320)^***^	337 (332, 343)^***^
HEI-2015 (100)	50.1 (49.5, 50.8)	49.7 (49, 50.3)	49.8 (49.2, 50.5)
HEI-1: Total fruits (5)	2.1 (2, 2.2)	2.2 (2.1, 2.3)	2.2 (2.1, 2.3)
HEI-2: Whole fruits (5)	2.1 (2, 2.2)	2 (1.9, 2.1)	2.1 (2, 2.2)
HEI-3: Total vegetables (5)	2.9 (2.8, 2.9)	2.8 (2.7, 2.8)	2.8 (2.7, 2.9)
HEI-4: Greens and beans (5)	1.5 (1.4, 1.6)	1.2 (1.1, 1.3)^***^	1.3 (1.2, 1.3)
HEI-5: Whole grains (10)	2.7 (2.6, 2.8)	2.3 (2.2, 2.4)^**^	2.4 (2.3, 2.5)
HEI-6: Dairy (10)	5.4 (5.2, 5.5)	2 (1.9, 2.1)^***^	3 (2.9, 3.1)^***^
HEI-7: Total protein foods (5)	4.1 (4.1, 4.1)	3.7 (3.6, 3.8)	3.8 (3.8, 3.9)
HEI-8: Seafood and plant protein (5)	2.2 (2.1, 2.3)	2.1 (2, 2.2)	2.1 (2, 2.2)
HEI-9: Fatty acids (10)	4.8 (4.7, 4.9)	6.9 (6.8, 7)^***^	6.3 (6.2, 6.4)
HEI-10: Refined grains (10)	6 (5.9, 6.1)	6 (5.9, 6.1)	6 (5.9, 6.1)
HEI-11: Sodium (10)	4.3 (4.2, 4.4)	4.9 (4.8, 5)^***^	4.8 (4.6, 4.9)^***^
HEI-12: Added sugars (10)	6.5 (6.3, 6.6)	5.9 (5.8, 6)	6 (5.9, 6.1)
HEI-13: Saturated fat (10)	5.7 (5.5, 5.8)	7.7 (7.6, 7.7)^***^	7.1 (7, 7.2)^***^
**Consumers of dairy fat only (*****n=*** **12,831)**			
NRF 9.3	402 (396, 407)	313 (307, 318)^***^	340 (335, 345)^***^
HEI-2015 (100)	50.4 (49.7, 51)	49.8 (49.2, 50.5)	50 (49.4, 50.7)
HEI-1: Total fruits (5)	2.1 (2.1, 2.2)	2.3 (2.2, 2.4)	2.3 (2.2, 2.3)
HEI-2: Whole fruits (5)	2.2 (2.1, 2.3)	2.1 (2, 2.2)	2.1 (2, 2.2)
HEI-3: Total vegetables (5)	2.9 (2.8, 2.9)	2.7 (2.7, 2.8)	2.8 (2.7, 2.9)
HEI-4: Greens and beans (5)	1.5 (1.4, 1.6)	1.2 (1.1, 1.2)^**^	1.2 (1.2, 1.3)^*^
HEI-5: Whole grains (10)	2.8 (2.7, 2.9)	2.4 (2.2, 2.5)^**^	2.5 (2.4, 2.6)
HEI-6: Dairy (10)	5.9 (5.8, 6.1)	2 (1.9, 2.1)^***^	3.2 (3, 3.3)^***^
HEI-7: Total protein foods (5)	4.1 (4, 4.1)	3.6 (3.6, 3.7)^*^	3.8 (3.7, 3.8)
HEI-8: Seafood and plant protein (5)	2.2 (2.1, 2.3)	2.1 (2, 2.2)	2.1 (2, 2.2)
HEI-9: Fatty acids (10)	4.4 (4.3, 4.5)	6.9 (6.8, 7)^***^	6.2 (6, 6.3)^***^
HEI-10: Refined grains (10)	6 (5.9, 6.1)	6.1 (6, 6.2)	6.1 (6, 6.2)
HEI-11: Sodium (10)	4.3 (4.2, 4.4)	5 (4.9, 5.2)^***^	4.9 (4.7, 5)^**^
HEI-12: Added sugars (10)	6.5 (6.4, 6.7)	5.9 (5.7, 6)	6 (5.9, 6.1)
HEI-13: Saturated fat (10)	5.3 (5.2, 5.5)	7.7 (7.6, 7.8)^***^	7 (6.9, 7.1)^***^

a Model 1 removes all dairy fat. Model 2 removes up to 5% dairy fat for each individual.

b Asterisks indicate statistical significance comparing whether Modeled changes are a 10% change from observed diets;

*** p < 0.001;

** 0.001 < p < 0.01;

* 0.01 < p < 0.05.

The removal of dairy fat led to lower NRF 9.3 scores; total HEI-2015 scores were not affected. At the HEI sub-score level, a reduction in points from dairy was compensated for by additional points from lower saturated fat and more PUFA. Higher sodium score reflected a lower content of sodium. For dairy consumers there was a small reduction in total protein score.

Table 7 shows observed and modeled mean NRF 9.3 and HEI-2015 overall and component scores following the replacement of dairy fat with foods not containing dairy fat. Among the total population, the NRF 9.3 and the HEI-2015 scores were not statistically different across observed and modeled diets, though NRF 9.3 scores tended to be lower, and HEI-2015 scores tended to be higher. Across HEI-2015 sub-scores, total vegetables, greens and beans, seafood and plant protein, fatty acid ratio, and saturated fat improved for Model 1 as compared to observed diets. Dairy and sodium worsened. Similar patterns were observed for Model 2, though effects were weaker. When limited to dairy fat consumers the HEI-2015 change for Model 1 was significant, increasing from 50.4 to 55.8 (out of 100). Total vegetables, greens and beans, seafood and plant proteins, fatty acid ratio and saturated fat improved, while sodium and dairy worsened. Whole fruits and total protein foods also increased when limiting the analysis to dairy fat consumers.

**Table 7:** Observed and modeled mean NRF 9.3 and HEI-2015 overall and component scores, following the replacement of dairy fat with PUFA/MUFA, NHANES 2013–2016.

	**Mean (95% Confidence Intervals [CI])**		
	**Observed**	**Model 1^**a**^ (swap)**	**Model 2^**b**^ (swap)**
**Total population (*****n=*** **15,260)**			
NRF 9.3	391 (385, 396)	362 (357, 367)	378 (372, 383)
HEI-2015 (100)	50.1 (49.5, 50.8)	54.7 (54.1, 55.3)	52.9 (52.3, 53.5)
HEI-1: Total fruits (5)	2.1 (2, 2.2)	2.3 (2.2, 2.3)	2.2 (2.1, 2.3)
HEI-2: Whole fruits (5)	2.1 (2, 2.2)	2.3 (2.3, 2.4)	2.3 (2.2, 2.4)
HEI-3: Total vegetables (5)	2.9 (2.8, 2.9)	3.3 (3.3, 3.4)^***^	3.2 (3.1, 3.2)
HEI-4: Greens and beans (5)	1.5 (1.4, 1.6)	2.1 (2, 2.1)^***^	1.9 (1.8, 2)^***^
HEI-5: Whole grains (10)	2.7 (2.6, 2.8)	2.8 (2.7, 2.9)	2.8 (2.6, 2.9)
HEI-6: Dairy (10)	5.4 (5.2, 5.5)	2.1 (2, 2.1)^***^	3.5 (3.4, 3.7)^***^
HEI-7: Total protein foods (5)	4.1 (4.1, 4.1)	4.5 (4.5, 4.5)^*^	4.4 (4.4, 4.4)
HEI-8: Seafood and plant protein (5)	2.2 (2.1, 2.3)	3.4 (3.3, 3.4)^***^	3 (2.9, 3.1)^***^
HEI-9: Fatty acids (10)	4.8 (4.7, 4.9)	7.9 (7.8, 8)^***^	6.5 (6.4, 6.6)^***^
HEI-10: Refined grains (10)	6 (5.9, 6.1)	6.1 (6, 6.2)	6.1 (6, 6.2)
HEI-11: Sodium (10)	4.3 (4.2, 4.4)	3.5 (3.4, 3.6)^***^	3.7 (3.6, 3.8)^***^
HEI-12: Added sugars (10)	6.5 (6.3, 6.6)	6.8 (6.7, 6.9)	6.7 (6.5, 6.8)
HEI-13: Saturated fat (10)	5.7 (5.5, 5.8)	7.7 (7.6, 7.8)^***^	6.7 (6.6, 6.8)^***^
**Consumers of dairy fat only (*****n=*** **12,831)**			
NRF 9.3	402 (396, 407)	369 (365, 374)	387 (382, 392)
HEI-2015 (100)	50.4 (49.7, 51)	55.8 (55.2, 56.3)^*^	53.7 (53.1, 54.2)
HEI-1: Total fruits (5)	2.1 (2.1, 2.2)	2.3 (2.3, 2.4)	2.3 (2.2, 2.3)
HEI-2: Whole fruits (5)	2.2 (2.1, 2.3)	2.5 (2.4, 2.6)^*^	2.3 (2.2, 2.4)
HEI-3: Total vegetables (5)	2.9 (2.8, 2.9)	3.4 (3.4, 3.5)^***^	3.2 (3.2, 3.3)^**^
HEI-4: Greens and beans (5)	1.5 (1.4, 1.6)	2.2 (2.2, 2.3)^***^	2 (1.9, 2.1)^***^
HEI-5: Whole grains (10)	2.8 (2.7, 2.9)	2.9 (2.8, 3)	2.9 (2.8, 3)
HEI-6: Dairy (10)	5.9 (5.8, 6.1)	2.1 (2, 2.1)^***^	3.8 (3.7, 3.9)^***^
HEI-7: Total protein foods (5)	4.1 (4, 4.1)	4.6 (4.5, 4.6)^***^	4.4 (4.4, 4.5)
HEI-8: Seafood and plant protein (5)	2.2 (2.1, 2.3)	3.6 (3.5, 3.6)^***^	3.2 (3.1, 3.2)^***^
HEI-9: Fatty acids (10)	4.4 (4.3, 4.5)	8.1 (8, 8.2)^***^	6.4 (6.3, 6.5)^***^
HEI-10: Refined grains (10)	6 (5.9, 6.1)	6.2 (6.1, 6.3)	6.1 (6, 6.2)
HEI-11: Sodium (10)	4.3 (4.2, 4.4)	3.3 (3.2, 3.4)^***^	3.6 (3.5, 3.8)^***^
HEI-12: Added sugars (10)	6.5 (6.4, 6.7)	6.9 (6.8, 7)	6.8 (6.7, 6.9)
HEI-13: Saturated fat (10)	5.3 (5.2, 5.5)	7.7 (7.6, 7.8)^***^	6.6 (6.5, 6.7)^***^

a Model 1 replaces all dairy fat. Model 2 replaces up to 5% dairy fat for each individual.

b Asterisks indicate statistical significance comparing whether Modeled changes are a 10% change from observed diets;

*** p < 0.001;

** 0.001 < p < 0.01;

* 0.01 < p < 0.05.

## Discussion

Replacing dairy fat with other forms of fat, such as a combination of PUFA/MUFA, should not be viewed as a simple exercise in regression modeling. Removing dairy fat from the diet means reducing the consumption of foods where dairy fat is the main source of fat or just an ingredient. In the NHANES sample, a mean reduction in 5% of energy from dairy fat meant removing about 65% of dairy foods from the diet. In the present NHANES 2013–2016 sample, dairy fat provided a mean of 5.6% of dietary energy with some differences by age and race/ethnicity.

Consumption patterns for dairy products vary widely across population subgroups. The consumption of fluid milk drops sharply with age; while the consumption of whole- vs. reduced-fat milk tracks socioeconomic status. Milk is more likely to be consumed at breakfast than at dinner. The consumption of dairy products can affect dietary energy density as well as the percent of fat energy in the diet. Realistic food-level substitution modeling needs to take these many social and behavioral considerations into account. Food-level substitutions need to be age-, meal-, and diet-specific.

Food level substitutions can never be perfect. First, many foods that contain dairy fat as an ingredient can also contain MUFA and PUFA. Conversely, many foods that contain MUFA/PUFA also contain meaningful amounts of saturated fat. Our rules were that foods that contained dairy fat could not be included in the substitution model, nor could foods that contained more saturated fat than MUFA+PUFA. Otherwise the substitution modeling was context specific with weighted substitution foods generated for 576 separate population subgroups

This food-level approach contrasts with simpler “fat swapping,” that is the swapping of regression coefficients in large scale observational studies (1). Those analyses did not take individual food choices into account but did adjust for age, BMI, energy intake, ethnicity, smoking, physical activity, alcohol consumption, hypertension and hypercholesterolemia. Replacing dairy fat with healthful carbohydrates via the same methods was also shown to reduce CVD risk (1). Nutrient-based dietary guidance is apt to ignore the difficulties inherent in replacing one food group for another, or the unintended nutritional consequences of removing some food groups from the diet altogether. Dairy products are a major source of fatty acids, micronutrients, and bioactive peptides.

The present analyses illustrate the difference between a food-based and a nutrient-based substitution model. The removal of the food sources of dairy fat from the diet led to dietary patterns that were significantly lower in calories and lower in some key micronutrients. Although saturated fat was significantly reduced, calcium and vitamins A, D, and B12 were significantly reduced as well. Diet quality measured using the nutrient-based NRF score was significantly reduced. These results held for partial (5%) or complete removal of dairy fat from the diet and held both for dairy fat consumers and for the whole US population.

The food-level replacement of non-dairy PUFA and MUFA for the missing dairy fat did not remedy the modeled lower intakes of some micronutrients. Measures of diet quality were equivocal. The NRF score showed a reduction in overall dietary nutrient density with the removal of dairy fat and no significant improvement when dairy fat was replaced with non-dairy PUFA and MUFA.

No major improvement in diet quality following PUFA/MUFA replacement was observed using the total HEI-2015 score, though for the dairy fat consumers there was an increase in HEI-2015 scores. This was surprising, given that the HEI-2015 penalizes intakes of saturated fat intake twice; first, as total intake and second, as the ratio of unsaturated to saturated fat. The saturated fat sub-score is very highly correlated with the fatty acid ratio sub-score; (*r* = 0.64) which is one of the strongest bivariate correlations among HEI-2015 components. Replacing saturated fat with unsaturated fat would practically guarantee higher HEI-2015 scores, given that this is how HEI-2015 was constructed. However, no expected improvement in HEI-2015 scores, the federal measure of diet quality, following the removal of dairy fat was observed.

Finally, the observation that modeled nutrient-for-nutrient substitutions were associated with a global reduction in CVD risks (1) seems to contrast with some of the new and emerging evidence for the beneficial impact of dairy fat (33–35). In particular, studies have suggested that dairy foods, particularly fermented dairy products, have neutral or inverse associations with CVD (36, 37). The current view is that overall dietary patterns, as opposed to selected single nutrients are the basis of healthy eating (36). Current analyses are turning to nutrients in the context of the food matrix.

Previous modeling studies did acknowledge the inability to evaluate any potential impact of the food matrix (1). In other words, different foods (butter, hard cheese) had a different impact on plasma biomarkers of CVD, at comparable intakes of total fat and saturated fat. Arguably, the food matrix is itself a simplification. Butter and hard cheese are consumed on different eating occasions and may be accompanied by different foods. The present study addressed the content of dairy fat consumption by taking into account eating occasion and the energy density of the food. Further work on links between diets and CVD risk needs to address food patterns in addition to selected single nutrients.

This study has limitations and strengths that are worth noting. Our results do not represent actual human behavior, which is complex and multi-factorial. We made an effort to build a sophisticated model that accounted for the context of consumption (e.g., meal type) and individual-characteristics (e.g., age), but we could not account for all such variables. However, our modeling strategy took many more factors into account than similar studies and we view this as an improvement. The use of large and nationally representative NHANES datasets ensured a large sample size, making the results highly generalizable and statistically stable. We assessed a number of different dietary outcomes, including individual macronutrients, micronutrients and summary measures of diet quality. A few important diet quality measures were not available, specifically *trans* fatty acids, which are not available in NHANES data. Lastly, a single 24 h recall allows us to examine the change in the population-mean but does not permit us to estimate in an unbiased manner the change in the proportion of individuals meeting or failing to meet a dietary threshold (e.g., calcium adequacy).

## Conclusion

Food-level modeling that is sensitive to dietary patterns points to some limitations of the theoretical “nutrient swapping” approach. Given that foods contain multiple nutrients, clean replacement of one nutrient for another may be simple in theory but very complex in real life. The Dietary Guidelines for Americans are increasingly adopting a food based approach with more attention paid to food patterns than to individual nutrients.

## Data Availability

The datasets for this study will not be made publicly available because the NHANES databases are publicly available and in the public domain.

## Author Contributions

CR and AD conceptualized and designed the study. CR developed the databases, carried out the analyses and produced summary tables. AD drafted the initial manuscript. All authors reviewed and revised the manuscript, and approved the final manuscript as submitted.

### Conflict of Interest Statement

AD has received grants, honoraria, and consulting fees from numerous food and beverage companies and other commercial and non-profit entities with interests in nutrient density of individual foods and of the total diet. CR has consulted for the Bell Institute (General Mills), Unilever, PepsiCo, and Nestle, all outside of the submitted work. Support for the current project was provided by the National Dairy Council.
